# Uncertainties in Calibration of an Optical Spectrometer for Measuring Isotope Ratios in Methane

**DOI:** 10.1021/acsmeasuresciau.5c00094

**Published:** 2026-02-10

**Authors:** Christopher Rennick, Emmal Safi, Aimee Hillier, Emily Hopkinson, Ruth E. Hill-Pearce, Tim Arnold

**Affiliations:** † National Physical Laboratory, Hampton Road, Teddington TW11 0LW, U.K.; ‡ Lund University Department of Physical Geography and Ecosystem Science, Sölvegatan 12, Lund, Skåne County SE 223 62, Sweden; § The University of Edinburgh School of GeoSciences, Edinburgh, Scotland UK EH9 3JW, U.K.

**Keywords:** methane, isotope ratio, isotopologue method, calibration, uncertainty, optical isotope ratio spectroscopy, OIRS

## Abstract

The stable isotope ratios of carbon (δ^13^C­(CH_4_)) and hydrogen (δ^2^H­(CH_4_)) in methane (CH_4_) from atmospheric air samples provide a tracer that can help distinguish the relative contribution of emission sources. These can be continuously measured at atmospheric monitoring stations by optical isotope ratio spectrometer (OIRS) instruments, providing data that is complementary to isotope ratio mass spectrometry (IRMS) measurements. OIRS instruments directly measure the amount fraction of the ^12^CH_4_, ^13^CH_4_, and ^12^CH_3_^2^H isotopologues, in contrast to the IRMS method of conversion to CO_2_ and H_2_, so they have different calibration needs related to reference materials (RMs) and analysis protocol. We use a single high-purity source of CH_4_, which has been isotopically characterized by IRMS, to produce two calibration RMs that bracket the sample in amount fraction. The isotope ratio measurement is calibrated via the isotopologue amount fraction, which is used to derive analytical expressions for the combined uncertainty. We test this approach using a 550 μmol mol^–1^ sample (representative of the amount fraction produced from air sampled by the NPL preconcentrator) prepared from a separate CH_4_ source. The combined standard uncertainty for the isotope ratio of this sample is 0.19 ‰ for δ^13^C­(CH_4_) and 1.1 ‰ for δ^2^H­(CH_4_), including uncertainty contributions from the isotopic assignment of the CH_4_ used for the RMs, their gravimetric preparation, and the spectrometer noise. The dominant contribution to this is from the uncertainty in isotopic assignment of the CH_4_ used in RM preparation. The next largest contribution to the uncertainty budget is the measurement noise, estimated from the Allan-Werle deviation, and the smallest contribution is from the preparation uncertainty in the total CH_4_ amount fraction in the RMs. We demonstrate that preparing the bracketing RMs from a common CH_4_ source results in correlations between isotopologue amount fractions and that neglecting this leads to an overestimation of the isotope ratio uncertainty.

## Graphical abstract

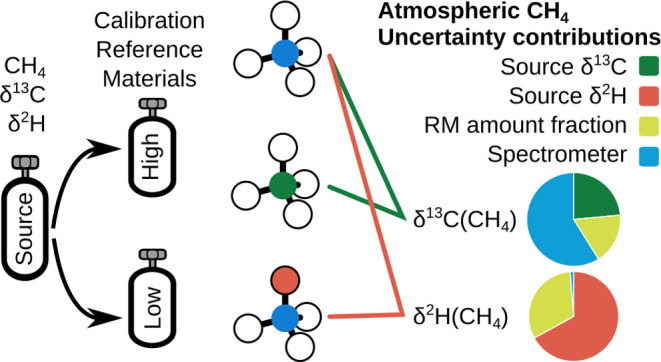

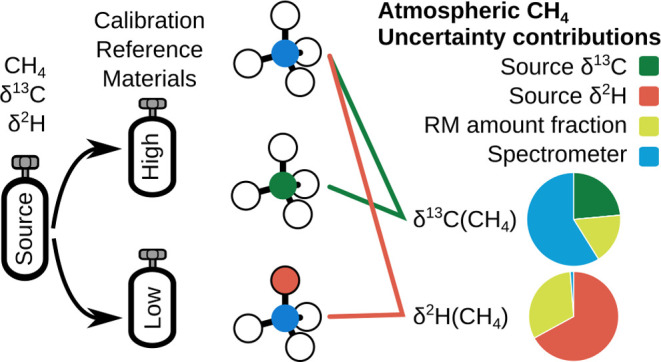

## 1 Introduction

The relative proportion of the stable isotopes of carbon and hydrogen in atmospheric methane (CH_4_) can be used as a tracer for the formation and loss processes and potentially disaggregate the relative emissions from source sectors.^1,2^ Long-term flask sampling at remote locations, followed by isotope ratio mass spectrometry (IRMS) analysis, has been used to measure the δ^13^C­(CH_4_) timeseries of the well-mixed background at weekly resolution.^3,4^ An IRMS instrument has also been deployed to tall tower sites to measure δ^13^C­(CH_4_) and δ^2^H­(CH_4_) at high frequency (one measurement every 20 min) for regional source attribution.^5,6^ IRMS instruments have well-established operating procedures to calibrate the instrument response with traceability to international standards of the stable isotope ratios ^13^C/^12^C and ^2^H/^1^H,^7,8^ while quantifying and minimizing instrumental artifacts such as contamination and drift.^9^

Optical techniques are widely used for amount fraction measurements at atmospheric monitoring stations, and the deployment of optical isotope ratio spectrometers (OIRS) for isotopic measurements is increasing. A variety of technologies are used in OIRS instruments, such as Fourier transform infrared spectroscopy (FTIR^10^), tunable diode laser absorption spectroscopy (TLDAS^11,12^ ), off-axis integrated cavity-output spectroscopy (OA-ICOS^13^), and cavity ringdown spectroscopy (CRDS^14^). The higher portability of OIRS compared to IRMS means that they have been used on mobile sampling platforms in Paris^15^ and Romania.^16^ OIRS instruments have also been deployed for measurement campaigns in more polluted areas, such as city centers^17^ and shale gas extraction fields.^18^ Commercial instruments making direct atmospheric measurements have lower precision than IRMS due to the low abundance of CH_4_; however, new instruments have been developed that use preconcentration to improve performance.^19,20^

Spectrometers report the amount fraction of the individual isotopologues in a sample (i.e., ^12^CH_4_, ^13^CH_4_, and ^12^CH_3_^2^H), so two approaches to calibration are possible that target either amount fraction or isotopologue ratio as the primary instrument response.^21^ Treating the isotope ratios δ^13^C­(CH_4_) and δ^2^H­(CH_4_) as the calibrated quantities requires characterizing the instrument using standard gases spanning a range of both isotope ratio and CH_4_ amount fraction.^14^ On the other hand, treating the isotopologue amount fractions as the calibrated quantity requires standard gases spanning the sample in amount fraction.^22^

The NPL preconcentrator-OIRS comprises a laser spectrometer with a custom preconcentration front-end that increases the amount fraction of CH_4_ from an atmospheric air sample—global monthly mean 1.933 μmol mol^–1^ in February 2025 ^23^—to more than 500 μmol mol^–1^ in a nitrogen matrix.^20^ Preconcentration is required for these air measurements to increase the signal-to-noise ratio of the spectrum to improve the precision of the reported amount fraction, but consequently the calibration gases must also match this higher amount fraction and nitrogen matrix. Calibration gases can, in principle, also be processed by the preconcentrator; however, the trapping cycle takes around 1 h, making this approach susceptible to drift in the instrument response in the time between measurement of the sample and calibration gas.

As was shown previously, calibration of the NPL preconcentrator-OIRS uses a single source of CH_4_ to prepare two calibration reference materials (RMs) that bracket the sample in amount fraction and then calibrate the spectrometer using the isotopologue amount fraction method.^20^ Here, we derive a combined uncertainty on the resulting δ^13^C­(CH_4_) and δ^2^H­(CH_4_) by rigorously propagating the uncertainties arising from the different arithmetic inputs to the ratio calculations. The analytical expressions are compared to a Monte Carlo method to verify these calculations and to determine the relative contributions to the uncertainty in measured isotope ratios from the measurement noise and RM preparation. The analytical expressions for uncertainty are also used to explore the sensitivity to the uncertainty in the RM amount fraction and show that this has a minimal effect under our conditions. We demonstrate that a two-point calibration strategy, using RMs prepared from a common CH_4_ source, is an effective and straightforward calibration method for OIRS. This calibration is referenced to the δ^13^C and δ^2^H isotope ratios of the CH_4_ standard, as measured by IRMS, and not an independent realization of the scale.

## 2 Experimental Section

### 2.1 Preparation of CH_4_ in N_2_ Mixtures

Two fossil sources have been used to prepare the CH_4_ in N_2_ mixtures. These have been assigned δ^13^C and δ^2^H by IRMS at BGC-isolab, measured after diluting to a near-ambient amount fraction in a synthetic air matrix. The isotope ratios are referenced to the JRAS-M16 scale, which is traceable to the NBS19/LSVEC and SMOW/SLAP primary standards for δ^13^C and δ^2^H, respectively.^24^ Note that the International Atomic Energy Agency (IAEA) identifies two carbon isotope ratio scales named VPDB and VPDB-LSVEC that differ between +0.14 and +0.30 ‰ for isotope ratio values around −45 ‰.^25–27^ The measurements here are on the VPDB-LSVEC scale because the CH_4_ has been measured against a working standard that is linked to the JRAS-M16 scale that is commonly used as the reference for the δ^13^C isotope ratio of CH_4_ in air.^5,6,24,28,29^ The uncertainties in the isotope ratios include contributions from the measurement of the sample and standard, the uncertainty from measuring in an air matrix, and the propagation to the primary scales.

The mixtures are prepared gravimetrically by first preparing a 2% CH_4_ in N_2_ intermediate mixture that is first added to the evacuated 10 L aluminum cylinders via a transfer vessel, followed by direct addition of N_2_. The intermediate mixture and transfer vessel are used to achieve a low uncertainty for the otherwise small mass additions. The gravimetric uncertainty in the CH_4_ amount fraction of the RMs is determined from the gravimetric preparation route, using the method outlined in the International Standard ISO 6142-1:2015 and implemented by the software package gravcalc2, which includes contributions from the uncertainty in mass, purity, and molar mass of the components.^30^ This does not include any component from the validation of the amount fraction. The composition of the mixtures is summarized in Table 1. The low and high calibration RMs and sample A are all prepared from the same pure CH4 source. Sample B is prepared from a different CH_4_ stock mixture at 2% in N_2_ prepared using a direct filling route rather than using a transfer vessel. The larger uncertainty in the amount fraction of the 2% CH_4_ in N_2_ parent results in a larger gravimetric amount fraction uncertainty for sample B.

**Table 1:** Isotopic Composition and Amount Fraction of CH_4_ in N_2_ Mixtures Used as Calibration RM and Samples^a^

**sample ID**	**description**	**amount fraction/μmol** mol^**–1**^	**δ** ^**13**^ **C/‰**	**δ** ^**2**^ **H/‰**
**D049795**	low calibration RM	500.480 ± 0.102	–39.27 ± 0.11	–197.8 ± 1.1
**D914016**	high calibration RM	620.134 ± 0.106	–39.27 ± 0.11	–197.8 ± 1.1
**10311**	sample A	549.57 ± 0.104	–39.27 ± 0.11	–197.8 ± 1.1
**NPL-3024**	sample B	550.00 ± 0.77	–51.87 ± 0.11	–190.7 ± 1.1

a The amount fraction is the gravimetric value with an uncertainty from preparation. The isotope ratios δ^13^C and δ^2^H are measured by IRMS and referenced to JRAS-M16. All uncertainties are given as *k* = 1 standard uncertainties.

### 2.2 OIRS Analyzer

The analyzer is an Aerodyne dual laser tunable infrared diode laser absorption spectrometer (TILDAS, Aerodyne Research Inc.) that measures a high-resolution spectrum of the major CH_4_ isotopologues ^12^CH_4_, ^13^CH_4_, and ^12^CH_3_^2^H in the 7.7 μm mid-IR band. This spectrometer uses a 0.5 L internal volume, 76 m optical path length cell that is first evacuated, then filled with the sample gas to a pressure of 37.5 mbar. The wavelength of the lasers is scanned through the absorption spectrum at a high rate (1.7 kHz repetition frequency) while the transmitted intensity is recorded by the photodiode. The spectra are averaged for 1 s, then fitted for amount fraction using a Voigt profile for the absorption lines using parameters from the HITRAN2020 molecular spectroscopic database^31^ and the measured cell temperature and pressure. The spectra from the two lasers are fitted separately: laser 1 is fitted for only ^12^CH_4_ and ^13^CH_4_ features in the region of interest, and laser 2 is fitted for ^12^CH_4_, ^12^CH_3_^2^H, H_2_O, and N_2_O as the latter two species have relatively strong absorption features in this region. The absence of H_2_O and N_2_O in the spectrum is used as a quality control for the preconcentration. Figure 1 shows an example of the 1 s raw spectrum for a 550 mmol mol^–1^ sample of CH_4_ in N_2_.

**Figure 1:**
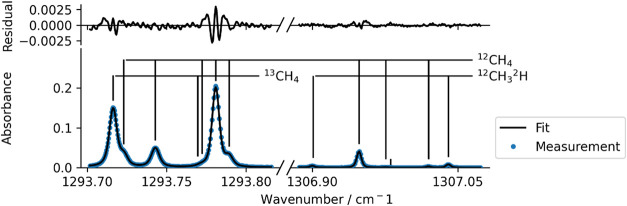
1 s average spectra are plotted (after baseline subtraction and conversion of the signal and reference to absorbance) as points in the lower plot overlaid with a solid line showing the fit used to derive an instrument response to each isotopologue. The markers above the spectrum indicate the isotopologues responsible for the dominant features. The residuals of the least-squares fit are shown in the top plot.

During an atmospheric measurement cycle the spectrometer is sequentially loaded with each calibration gas mixture, followed by the preconcentrated sample. The amount fraction of the CH_4_ isotopologues is recorded to a data file at one second intervals for processing. The spectral fit from laser 1 is used for the ^12^CH_4_ measurement as the absorption is much stronger than in laser 2, giving a better signal-to-noise ratio. This means that the measurements for δ^13^C­(CH_4_) use data from only laser 1, and the measurements for δ^2^H­(CH_4_) require data from both lasers. Note that the spectrometer reports a quantity for each isotopologue that is scaled by the isotopologue abundance, specified in the HITRAN2020 spectroscopic database (*X*_211_(HITRAN2020) = 0.988274, *X*_311_(HITRAN2020) = 1.11031 × 10^–2^, and *X*_212_(HITRAN2020) = 6.15751 × 10^–4^).^31^ This scaling has the effect of making the instrument response a similar magnitude as the sample amount fraction in nmol mol^–1^. The response (and its uncertainty) is treated here as a unitless instrument signal in the calibration procedure using bracketing standards so that the details of the spectroscopy are not considered.

### 2.3 OIRS Measurements

A complete OIRS measurement cycle involves measurement of the two calibration RMs, followed by the sample. Each gas is loaded by first evacuating the spectrometer cell to below 0.15 mbar using an oil-free scroll pump, then opening the filling valve to flush the gas manifold and spectrometer cell with the sample for a further 30 s. The filling valve is then closed, and the spectrometer is evacuated a second time. The gas is loaded into the spectrometer by first filling an evacuated 50 mL volume stainless steel cylinder to a target pressure of 368 mbar by closing the inlet solenoid valve under computer control, which is triggered by a high-resolution pressure sensor. The cell evacuation valve is closed, then the inlet valve is opened, allowing the contents of the 50 mL volume to equilibrate with the evacuated spectrometer cell, reaching a final pressure of 24 mbar. This filling sequence flushes the gas lines and spectrometer with the sample, minimizing carry-over, and provides a high degree of repeatability to the cell pressure. A 20 s delay allows the temperature to stabilize, and then data is logged at 1 s intervals for 100 s.

The 100 s measurement duration is chosen from the minimum in the Allan-Werle deviation plot, which is shown for one sample in Figure 2a.^32^ The one second measurements also confirm that there is minimal correlation between the instrument response to each isotopologue. Figure 2b plots these as scatter pair-plots of the three isotopologues measured in each 1 s interval. The Pearson product-moment correlation coefficients are *r*(*R*_211_, *R*_311_) = 0.12 and *r*(*R*_211_, *R*_212_) = 0.10, which are below the level of statistically significant correlation for 100 samples (*r*_crit_ = 0.20 at *p* = 0.05). The scatter plots show clearly that there is little correlation between the reported isotopologue amount fractions. The data in the lower plot are measured by the two separate lasers (^12^CH_4_ from laser 1 and ^12^CH_3_^2^H vs from laser 2). The data in the upper plot (^12^CH_4_ vs ^13^CH_4_) are both measured using laser 1 and show a similar scatter that indicates negligible systematic effects between the two species fitted from scanning a single laser.

**Figure 2:**
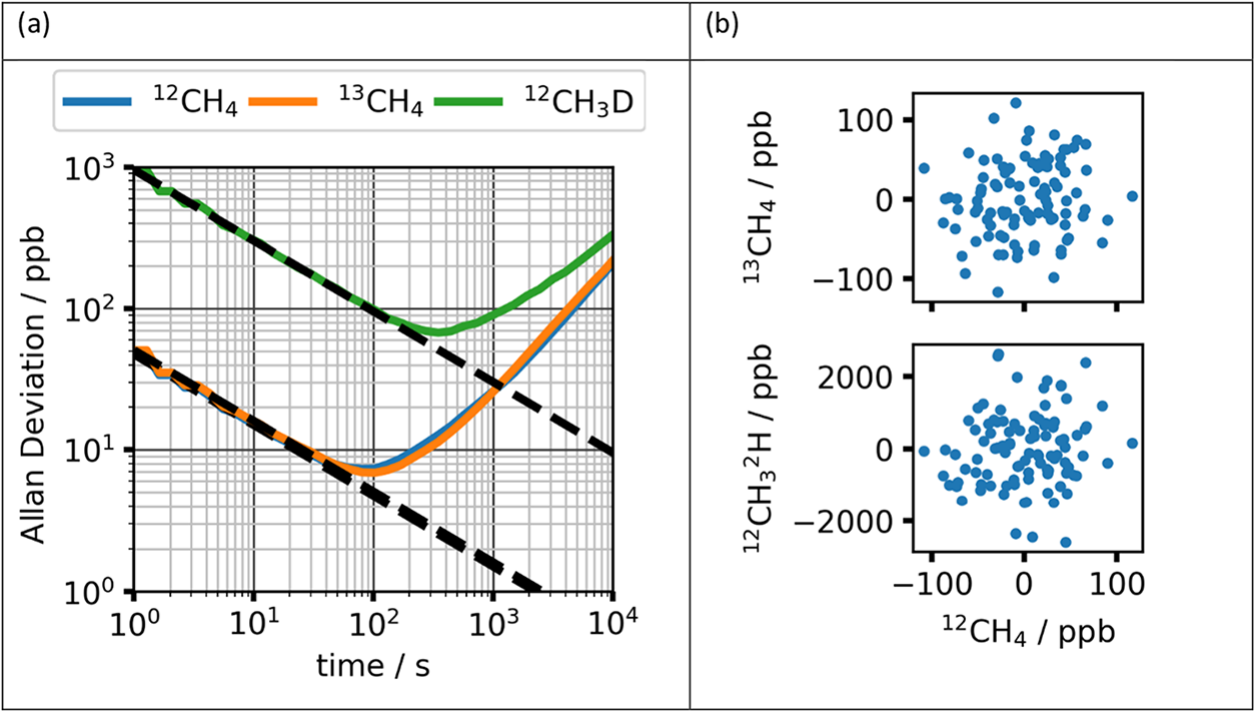
(a) Allan Deviation plotted as a function of averaging time for the spectrometer response to the low calibration RM. The dashed line represents the expected trend with integration time for random noise. (b) Correlation plot between the pair of isotopologue instrument responses from each 1 s spectrum over the 100 s measurement interval. Measurements are plotted as the difference from the mean for clarity. The axis units are the values reported by the instrument that are scaled by the natural abundance stated in the HITRAN database. The *x*-axis is the same for both plots, as ^12^CH_4_ is measured in both cases using laser 1.

The calibration method using two RMs that bracket the sample in amount fraction assumes that the spectrometer is linear in response, and that the details of deriving an amount fraction from the spectrum can be ignored, including the HITRAN scaling factor applied to the instrument response, as described in Section 2.2. The linearity of the spectrometer response to the amount fraction has been confirmed previously using mixtures up to 750 μmol mol^–1^. The HITRAN scaling factor, arising from the spectrum fitting parameters and described in Section 2.2, appears in the numerator and denominator of eq 18, so it is canceled from the calculation.

Here, we perform a calibration under the local conditions by measuring the sample and RMs as close in time as possible. During repeated cycles, the RMs are measured before every sample so that the instrument responses used in the calibration are local in time. Over durations much longer than 1000 s, systematic effects increasingly cause drift in the response due to the influence on the spectroscopy, such as the cell temperature and laser scanning conditions.

## 3 Calibration

The isotopologue calibration method has been applied to CO_2_ FTIR spectrometers^10,22,33^ and tunable diode laser absorption spectrometers measuring CH_4_ and CO_2_.^20,34^ In general, this approach calibrates the instrument response to each isotopologue amount fraction separately using a pair of RMs that bracket the sample in amount fraction. The calibrated isotope ratio is then given by the ratio of the calibrated isotopologue amount fractions.

In the following subsections, we outline the steps to the method and develop analytical expressions for the uncertainty of the output quantity of each step. The calibration scheme is treated as a multistep measurement model, where the output quantities of one step are input quantities for the next step, and the uncertainty estimate follows the Guide to the Expression of Uncertainty in Measurement (GUM).^35^ The combined uncertainty of an output quantity, *u*_*c*_(y), is expressed in terms of the uncertainties of each of the *N* input quantities *u*(*x*_*i*_):

1 $$u_{c}^{2}\left(y\right)=\sum_{i=1}^{N}{\left(C_{i}u\left(x_{i}\right)\right)}^{2}+2\sum_{i=1}^{N}\sum_{j=i+1}^{N}C_{i}C_{j}u\left(x_{i},x_{j}\right)$$

This expression includes the covariance of a pair of input quantities *u*(*x*_*i*_,*x*_*j*_), as it will be shown that some quantities are correlated. The sensitivity coefficients *C*_*i*_ = ∂*f*/∂*x*_*i*_ are the partial derivatives of the measurement equation with respect to the input quantity.

### 3.1 Isotopologue Abundance

The isotope ratios δ^13^C and δ^2^H of the pure CH_4_ that is used to prepare the calibration RMs are assigned by IRMS measurement. These measured isotope ratios are used to calculate the amount fraction of the isotopologues in the RMs by first calculating the isotopologue abundance, which is the normalized quantity of each isotopologue relative to all CH_4_ isotopologues in the pure CH_4_ parent (the abundances of all isotopologues sum to 1). As both RMs use the same CH_4_ source, this calculation only needs to be performed once. The stable isotopes of carbon (^12^C and ^13^C) and hydrogen (^1^H and ^2^H) combine to form ten isotopologues of CH_4_, and we measure the three most common ^12^CH_4_, ^13^CH_4_, and ^12^CH_3_^2^H. Assuming a statistical distribution of multiply substituted isotopologues—i.e., no clumping—the ratios *r*_13_ = *n*(^13^C)/*n*(^12^C) and *r*_2_ = *n*(^2^H)/*n*(^1^H) are calculated from δ^13^C and δ^2^H:

2 $$r_{13}=r_{13}^{\mathrm{VPDB}}\left(1+\delta^{13}C\right)$$

3 $$r_{2}=r_{2}^{\mathrm{VSMOW}}\left(1+\delta^{2}H\right)$$

where *r*_13_^VPDB^ = 0.011180 ± 0.000028 and *r*_2_^VSMOW^ = 0.00015575 ± 0.00000008 are the reference isotope ratios.^7,8^ An updated value for *r*_13_^VPDB^ has been endorsed by the CIAAW in 2024, which will replace the recommended value.^27,36^ The uncertainties on these reference ratios are given at the 95% confidence level for reference but are not propagated through this calibration because we do not include the uncertainty in defining the scale. This will be discussed in more detail in Section 5.2.

Applying the notation used for CO_2_ in^22^ to CH_4_, the abundance of the three major isotopologues is then calculated from

4 $$X_{211}=\frac{1}{R_{\mathrm{sum}}}$$

5 $$X_{311}=\frac{r_{13}}{R_{\mathrm{sum}}}$$

6 $$X_{212}=\frac{4r_{2}}{R_{\mathrm{sum}}}$$

where *R*_sum_ = (1 + *r*_13_)­(1 + *r*_2_)^4^ and the isotopologues are labeled by the AFGL notation *X*_211_ = *X*(^12^CH_4_), *X*_311_ = *X*(^12^CH_4_), and *X*_212_ = *X*(^12^CH_3_^2^H).^31^

#### 3.1.1 Isotopologue Abundance Uncertainty

The isotope ratios δ^13^C and δ^2^H are assigned by IRMS measurement of a sample of the source material and have associated measurement uncertainties *u*(δ^13^C) and *u*(δ^2^H) that are propagated to an uncertainty in isotopologue abundance. These isotope ratio assignments are measured independently, so covariance is zero and the uncertainty in isotopologue abundance is given by the first term in eq 1:

7 $$u^{2}\left(X_{211}\right)={\left(-\frac{r_{13}^{\mathrm{VPDB}}}{R_{\mathrm{sum}}\left(1+r_{13}\right)}\right)}^{2}u^{2}\left(\delta^{13}C\right)+{\left(-\frac{4r_{2}^{\mathrm{VSMOW}}}{R_{\mathrm{sum}}\left(1+r_{2}\right)}\right)}^{2}u^{2}\left(\delta^{2}H\right)$$

$$u^{2}\left(X_{311}\right)={\left(\frac{r_{13}^{\mathrm{VPDB}}}{R_{\mathrm{sum}}\left(1+r_{13}\right)}\right)}^{2}u^{2}\left(\delta^{13}C\right)+{\left(-\frac{4r_{13}r_{2}^{\mathrm{VSMOW}}}{R_{\mathrm{sum}}\left(1+r_{2}\right)}\right)}^{2}u^{2}\left(\delta^{2}H\right)$$ 8

9 $$u^{2}\left(X_{212}\right)={\left(-\frac{4r_{2}r_{13}^{\mathrm{VPDB}}}{R_{\mathrm{sum}}\left(1+r_{13}\right)}\right)}^{2}u^{2}\left(\delta^{13}C\right)+{\left(\frac{4r_{2}^{\mathrm{VSMOW}}}{R_{\mathrm{sum}}}\left(1-\frac{4r_{2}}{\left(1+r_{2}\right)}\right)\right)}^{2}u^{2}\left(\delta^{2}H\right)$$

The sensitivity coefficients are derived by applying the chain rule to eqs 2–6.

#### 3.1.2 Isotopologue Abundance Covariance

The calculated abundance of each isotopologue depends on both isotope ratios and *R*_sum_; therefore, the uncertainty will be correlated. Supplement 2 to the GUM describes propagation of uncertainty through a multivariate model, in which a set of output quantities ***y*** = (*y*_1_, *y*_2_, ..., *y*_m_) is calculated from a set of input quantities ***x*** = (*x*_1_, *x*_2_, ..., *x*_*N*_) using a set of multivariate functions ***f*** = (*f*_1_, *f*_2_, ..., *f*_m_). The covariance matrix of the output quantities is given by ***U***_***y***_ = ***C***_***x***_ ***U***_***x***_ ***C***_***x***_, where ***U***_***x***_ is a matrix of input quantity covariances and ***C***_***x***_ is a matrix of sensitivity coefficients *C*_*ij*_ = ∂*f_j_*/∂*x*_*i*_.^37^ This can be generalized to calculate the correlation coefficient if the input quantities are uncorrelated as *r*(*y*_*k*_,*y*_*l*_) = Σ_*i*_ *h*_*i*_(*y*_*k*_)*h*_*i*_(*y*_*l*_) and for correlated input quantities as *r*(*y*_*k*_,*y*_*l*_) = Σ_*i*_Σ_*j*_ *h*_*i*_(*y*_*k*_)*h*_*j*_(*y*_*l*_)*r*(*x*_*i*_,*x*_*j*_), where $h_{i}\left(y_{j}\right)=C_{\mathrm{ij}}\frac{u\left(x_{i}\right)}{u\left(y_{j}\right)}$ is the uncertainty contribution coefficient.^38,39^

The isotopologue abundance covariances for the pairings relevant to the two isotope ratios are

10 $$u\left(X_{211},X_{311}\right)=-{\left(\frac{r_{13}^{\mathrm{VPDB}}}{R_{\mathrm{sum}}\left(1+r_{13}\right)}\right)}^{2}u^{2}\left(\delta^{13}C\right)+r_{13}{\left(\frac{4r_{2}^{\mathrm{VSMOW}}}{R_{\mathrm{sum}}\left(1+r_{2}\right)}\right)}^{2}u^{2}\left(\delta^{2}H\right)$$

and

$$u\left(X_{211},X_{212}\right)=4r_{2}{\left(\frac{r_{13}^{\mathrm{VPDB}}}{R_{\mathrm{sum}}\left(1+r_{13}\right)}\right)}^{2}u^{2}\left(\delta^{13}C\right)-\left(1-3r_{2}\right){\left(\frac{4r_{2}^{\mathrm{VSMOW}}}{R_{\mathrm{sum}}\left(1+r_{2}\right)}\right)}^{2}u^{2}\left(\delta^{2}H\right)$$ 11

The third covariance *u*(*X*_212_, *X*_311_) is between the abundance of the ^13^CH_4_ and ^12^CH_3_^2^H isotopologues. The amount fraction of these two isotopologues is not used together in the isotope ratio calculations.

### 3.2 Amount Fraction

The calibration RMs are prepared by gravimetric dilution of the CH_4_ parent gas in a N_2_ matrix. The isotopologue amount fraction is the product of the isotopologue abundance and the CH_4_ amount fraction that is assigned gravimetrically:

12 $$Y_{211}^{\mathrm{low}}=X_{211}Y^{\mathrm{low}}$$

where the subscript 211 represents the isotopologue, the superscript “low” denotes the mixture identity. This calculation is repeated for all isotopologues (211, 311, 212) for both bracketing mixtures (low, high). The gravimetric total CH_4_ amount is denoted *Y* ^low^, i.e., without an isotopologue label.

#### 3.2.1 Amount Fraction Uncertainty

The combined uncertainty for these amount fractions contains a contribution from the isotopologue abundance and the total CH_4_ amount fraction:

13 $$u^{2}\left(Y_{211}^{\mathrm{low}}\right)={\left(u\left(X_{211}\right)Y^{\mathrm{low}}\right)}^{2}+{\left(u\left(Y^{\mathrm{low}}\right)X_{211}\right)}^{2}$$

This is also repeated for each isotopologue in both RMs. The CH_4_ amount fraction *Y* ^low^ is of all isotopologues in the parent gas, and the gravimetric determination requires an estimate of the molar mass, which depends on the isotope ratio. A nominal isotope ratio is used for gravimetry, and this is accounted for in the uncertainty u­(*Y* ^low^).

#### 3.2.2 Amount Fraction Covariance

The covariance between isotopologue abundances is propagated to the amount fractions using the GUM multivariate model approach:

14 $$u\left(Y_{211}^{\mathrm{low}},Y_{311}^{\mathrm{low}}\right)={\left(Y^{\mathrm{low}}\right)}^{2}u\left(X_{211},X_{311}\right)+X_{211}X_{311}u^{2}\left(Y^{\mathrm{low}}\right)$$

15 $$u\left(Y_{211}^{\mathrm{low}},Y_{212}^{\mathrm{low}}\right)={\left(Y^{\mathrm{low}}\right)}^{2}u\left(X_{211},X_{212}\right)+X_{211}X_{212}u^{2}\left(Y^{\mathrm{low}}\right)$$

Preparation of the two calibration RMs from the same parent CH_4_ will also result in covariance between the amount fraction of the isotopologue in each, e.g., the amount fraction of ^12^CH_4_ will be correlated in the low and high RM. This will have a contribution to the uncertainty of the amount fraction in the sample when this pair is used to bracket the sample in amount fraction. The covariance for ^12^CH_4_ is

16 $$u\left(Y_{211}^{\mathrm{low}},Y_{211}^{\mathrm{high}}\right)=Y^{\mathrm{low}}Y^{\mathrm{high}}u^{2}\left(X_{211}\right)$$

and is calculated similarly for the other isotopologues.

### 3.3 Spectrometer Response Calibration

The calculations for the isotopologue amount fractions, uncertainty, and covariance in the preceding sections need only to be performed once for the pure CH_4_ used to prepare the RMs. These RMs are then used to calibrate instrument response to the sample as a two-point interpolation, which assumes a linear spectrometer response over the expected sample range. It is convenient to represent this two-point interpolation by the weighted average of the amount fraction of the low and high calibration mixtures:

17 $$Y_{211}^{\mathrm{samp}}=\left(1-\alpha_{211}\right)Y_{211}^{\mathrm{low}}+\alpha_{211}Y_{211}^{\mathrm{high}}$$

The weighting for each isotopologue (here ^12^CH_4_) is given by the instrument response *R*_211_ to the sample and the standards:

18 $$\alpha_{211}=\frac{R_{211}^{\mathrm{samp}}-R_{211}^{\mathrm{low}}}{R_{211}^{\mathrm{high}}-R_{211}^{\mathrm{low}}}$$

#### 3.3.1 Uncertainty of Spectrometer Calibration

The combined uncertainty in the sample amount fraction can then be calculated using the weighting α_211_ from eq 18, the uncertainty, and the covariance in amount fraction for the standards:

19 $$u^{2}\left(Y_{211}^{\mathrm{samp}}\right)={\left(Y_{211}^{\mathrm{high}}-Y_{211}^{\mathrm{low}}\right)}^{2}u^{2}\left(\alpha_{211}\right)+{\left(1-\alpha_{211}\right)}^{2}u^{2}\left(Y_{211}^{\mathrm{low}}\right)+\alpha_{211}^{2}u^{2}\left(Y_{211}^{\mathrm{high}}\right)+2\alpha \left(1-\alpha \right)u\left(Y_{211}^{\mathrm{low}},Y_{211}^{\mathrm{high}}\right)$$

The combined uncertainty of the weighting factor is

20 $$u^{2}\left(\alpha_{211}\right)=\frac{1}{{\left(R_{211}^{\mathrm{high}}-R_{211}^{\mathrm{low}}\right)}^{2}}u^{2}\left(R_{211}^{\mathrm{samp}}\right)+\frac{{\left(\alpha_{211}-1\right)}^{2}}{{\left(R_{211}^{\mathrm{high}}-R_{211}^{\mathrm{low}}\right)}^{2}}u^{2}\left(R_{211}^{\mathrm{low}}\right)+\frac{{\left(\alpha_{211}\right)}^{2}}{{\left(R_{211}^{\mathrm{high}}-R_{211}^{\mathrm{low}}\right)}^{2}}u^{2}\left(R_{211}^{\mathrm{high}}\right)$$

where *u* ^2^(*R*_211_^samp^), etc., are the measurement uncertainties of the spectrometer to the ^12^CH_4_ isotopologue. The other isotopologue amount fractions are calculated in the same way.

#### 3.3.2 Covariance in Calibrated Isotopologue Amount Fractions

The absorption features for each isotopologue recorded by the high-resolution diode laser spectrometer are well separated with little overlap in the spectrum, so that it is assumed that there is no covariance arising from the raw measurement data. This is confirmed by the low correlation coefficient between isotopologue absorbance in the raw data, as shown in the Results Section. The covariance between calibrated isotopologue amount fractions results from preparation of the RMs and is calculated as the weighted average of the covariance in the bracketing standards:

21 $$u\left(Y_{211}^{\mathrm{samp}},Y_{311}^{\mathrm{samp}}\right)=\left(1-\alpha_{211}\right)\left(1-\alpha_{311}\right)u\left(Y_{211}^{\mathrm{low}},Y_{311}^{\mathrm{low}}\right)+\alpha_{211}\alpha_{311}u\left(Y_{211}^{\mathrm{high}},Y_{311}^{\mathrm{high}}\right)$$

22 $$u\left(Y_{211}^{\mathrm{samp}},Y_{212}^{\mathrm{samp}}\right)=\left(1-\alpha_{211}\right)\left(1-\alpha_{212}\right)u\left(Y_{211}^{\mathrm{low}},Y_{212}^{\mathrm{low}}\right)+\alpha_{211}\alpha_{212}u\left(Y_{211}^{\mathrm{high}},Y_{212}^{\mathrm{high}}\right)$$

### 3.4 Calculation of Isotopologue Ratio

The final step is to calculate the isotopologue ratio from the ratio of the calibrated isotopologue amount fractions, relative to the standard isotope ratios:

23 δ13C(CH4)=Y311sampY211sampr13VPDB−1

24 δ2H(CH4)=Y212sampY211samp4r2VSMOW−1

#### 3.4.1 Uncertainty in Isotope Ratio

The combined uncertainty for the isotope ratio includes both the uncertainty in isotopologue amount fraction and the covariance in isotopologue amount fraction between the RMs.:

$$\begin{array}{lll} u^{2}\left(\delta^{13}C\left({\mathrm{CH}}_{4}\right)\right) & = & {\left(-\frac{Y_{311}^{\mathrm{samp}}}{{\left(Y_{211}^{\mathrm{samp}}\right)}^{2}r_{3}^{\mathrm{VPDB}}}\right)}^{2}u^{2}\left(Y_{211}^{\mathrm{samp}}\right)\\ & + & {\left(\frac{1}{Y_{211}^{\mathrm{samp}}r_{13}^{\mathrm{VPDB}}}\right)}^{2}u^{2}\left(Y_{311}^{\mathrm{samp}}\right)\\ & + & 2\left(-\frac{Y_{311}^{\mathrm{samp}}}{{\left(Y_{211}^{\mathrm{samp}}\right)}^{2}r_{13}^{\mathrm{VPDB}}}\right)\left(\frac{1}{Y_{211}^{\mathrm{samp}}r_{13}^{\mathrm{VPDB}}}\right)u\left(Y_{211}^{\mathrm{samp}},Y_{311}^{\mathrm{samp}}\right) \end{array}$$ 25

$$\begin{array}{lll} u^{2}\left(\delta^{2}H\left({\mathrm{CH}}_{4}\right)\right) & = & {\left(-\frac{Y_{212}^{\mathrm{samp}}}{{4(Y_{211}^{\mathrm{samp}})}^{2}r_{2}^{\mathrm{VSMOW}}}\right)}^{2}u^{2}\left(Y_{211}^{\mathrm{samp}}\right)\\ & + & {\left(\frac{1}{4Y_{211}^{\mathrm{samp}}r_{2}^{\mathrm{VSMOW}}}\right)}^{2}u^{2}\left(Y_{212}^{\mathrm{samp}}\right)\\ & + & 2\left(-\frac{Y_{212}^{\mathrm{samp}}}{4{\left(Y_{211}^{\mathrm{samp}}\right)}^{2}r_{2}^{\mathrm{VSMOW}}}\right)\left(\frac{1}{4Y_{211}^{\mathrm{samp}}r_{2}^{\mathrm{VSMOW}}}\right)u\left(Y_{211}^{\mathrm{samp}},Y_{212}^{\mathrm{samp}}\right) \end{array}$$ 26

## 4 Results and Combined Uncertainty from Calibration

The sources of uncertainty in the calibrated isotope ratio of a sample are the isotope ratio of the CH_4_ used to prepare the calibration RMs, the amount fraction of the RMs, and the instrument response; these are summarized in Table 2.

**Table 2:** Input Quantities and Standard Uncertainty Sources for Measurement of Sample B (NPL-3024)^a^

**parameter**	**source**	**uncertainty** ***u*** **(** ***x*** **)**	**relative uncertainty** (%)
δ^13^C	isotope ratio of calibration CH_4_ assigned by IRMS	0.11 ‰	0.28
δ^2^H		1.1 ‰	0.54
*Y* ^low^	amount fraction of gravimetrically prepared RM	0.102 μmol mol^–1^	0.02
*Y* ^high^		0.106 μmol mol^–1^	0.02
*R*_211_^low^	instrument noise and drift, estimated from Allan-Werle deviation	49 nmol mol^–1^	0.01
*R*_311_^low^		45 nmol mol^–1^	0.01
*R*_212_^low^		91 nmol mol^–1^	0.02
*R*_211_^high^		42 nmol mol^–1^	0.01
*R*_311_^high^		57 nmol mol^–1^	0.01
*R*_212_^high^		75 nmol mol^–1^	0.02
*R*_211_^samp^		49 nmol mol^–1^	0.01
*R*_311_^samp^		50 nmol mol^–1^	0.01
*R*_212_^samp^		67 nmol mol^–1^	0.02

a The instrument response uncertainty given in this table is a representative example from a single run, as this varies from run-to-run and is calculated as described in the text.

The uncertainties in δ^13^C and δ^2^H for the CH_4_ used to prepare the calibration RMs are provided by the IRMS measurement. We do not add any uncertainty in the isotope ratio from fractionation during RM preparation as CH_4_ mixtures are known to be stable with respect to the amount fraction in treated cylinders, which indicates negligible wall losses. No fractionation has been observed in the preparation and storage of CH_4_ in air standards at ambient and elevated amount fractions.^24,40,41^ The uncertainty in the CH_4_ amount fraction of the RMs is determined from the gravimetric preparation, as described in Section 2.1.

The spectrometer measurement uncertainty used in the calculations—*u* ^2^(*R*_211_^low^), *u* ^2^(*R*_211_^high^), and *u* ^2^(*R*_211_^samp^) in eq 20—is larger than the minimum Allan-Werle deviation that can be seen in Figure 2a. This is because a complete measurement cycle requires the measurement of the three different mixtures, and this process takes around 620 s. For ^12^CH_4_ and ^13^CH_4_, we use the standard deviation of the 100 s data set, which happens to be approximately equal to Allan-Werle deviation at 1000 s, as a conservative estimate of the measurement uncertainty to include these effects over the complete measurement cycle. ^12^CH_3_^2^H is measured using the second laser, so we use the Allan-Werle deviation at 1000 s because the minimum occurs later than the major isotopologues. The flushing procedure purges the cell before filling, and the spectrometer measures and accounts for sample pressure during the fit, so these contributions are smaller than the Allan-Werle deviation.

Following the steps described in Section 3, the calibrated isotope ratios for the sample gases are given in Table 3. The combined standard uncertainties are calculated using the analytical expressions defined in Section 3. The calibrated isotope ratio and uncertainty for a preconcentrated air sample are also given. However, this is the measurement of the post-preconcentrator CH_4_, so it does not account for fractionation in the preconcentrator.^42^

**Table 3:** Calibrated Isotope Ratio for Two Samples Prepared from Pure CH_4_ and One Air Sample from the Boreas Preconcentrator Measured by OIRS^a^ [Sec sec3]

	**δ** ^**13**^ **C(CH**_**4**_ **)/‰**	**expected/‰**	**δ** ^**2**^ **H(CH**_**4**_ **)/‰**	**expected/‰**
**sample A**	–39.33 ± 0.17	–39.27 ± 0.11	–197.3 ± 1.1	–197.8 ± 1.1
**sample B**	–51.99 ± 0.19	–51.87 ± 0.11	–190.5 ± 1.1	–190.7 ± 1.1

a The combined standard uncertainty is calculated from the analytical expressions in Section 3.

The difference between the measured and expected δ^13^C­(CH_4_) measurements is −0.05 ‰ ± 0.20 ‰ and −0.12 ‰ ± 0.22 ‰ for samples A and B, respectively (the uncertainty is the square root of the sum of the individual uncertainties squared). The difference between the calibrated and expected δ^2^H­(CH_4_) measurements is 0.4 ‰ ± 1.5 ‰ and 0.2 ‰ ± 1.5 ‰ for samples A and B, respectively. The calibrated measurements are in good agreement with the expected values within the measurement uncertainties. It is notable that for δ^2^H­(CH_4_), the calibrated uncertainty is the same as the uncertainty in the CH_4_ used to prepare the RMs, implying that the other parameters have a negligible contribution to the combined uncertainty. The next section will quantify these contributions.

## 5 Contributions to the Calibration Uncertainty

Uncertainties in the input quantities have the potential to introduce a systematic bias to repeated measurements, so it is important to quantify the sensitivity to these quantities.

### 5.1 Validation by Monte Carlo Method

The Monte Carlo method (MCM) is a numerical approach to calculating the distribution of output parameters by repeated application of the measurement equations to input parameters that are randomly selected from a probability distribution. Comparing the standard deviation and covariance of the MCM results with the uncertainties and covariances calculated using the GUM method can validate the assumptions in the GUM framework of a linear measurement model and a normal probability distribution.^43^

Applying the MCM to the calculation of δ^13^C­(CH_4_) and δ^2^H­(CH_4_) of sample B for 10^4^ iterations, then taking the standard deviation of the output gives a quantity that agrees with the GUM method to better than two significant figures. Likewise, the uncertainties of the intermediate quantities, such as isotopologue amount fractions in the low and high RMs, and their covariances, also agree well between MCM and the GUM framework.

### 5.2 Uncertainty Budget

The uncertainty budget gives the contribution of each input quantity to the uncertainty of the output quantity and can be used to identify the dominant contributions. In principle, the GUM framework can be applied to derive the uncertainty budget. However, expressing the sensitivity coefficients as the partial derivatives of all steps of the measurement equations in Section 3 becomes unwieldy. Instead, the results of the MCM are used to approximate the sensitivity coefficients using the correlation coefficient between each input parameter and the output.^44^ The relative contribution to the total uncertainty is calculated from the normalized Spearman correlation coefficient between each parameter and the isotope ratio *S*_*i*_ = ρ^2^(*X*_*i*_,δ)/Σ_*j*_ρ^2^(*X*_*j*_,δ), where δ represents either δ^13^C­(CH_4_) or δ^2^H­(CH_4_). The contribution to the total uncertainty for samples A and B is plotted in Figure 3 as *u*(δ)√*S*_*i*_ for each parameter. This is the contribution of each parameter to the total uncertainty (in permille, ‰) so that the sum of the squared values is approximately equal to the squared total uncertainty (i.e., each is equivalent to the *C*_*i*_ *u*(*x*_*i*_) term in eq 1). The results for sample A, sample B, and a sample with the nominal isotopic composition of atmospheric air are plotted. The air sample is discussed in the next section.

**Figure 3:**
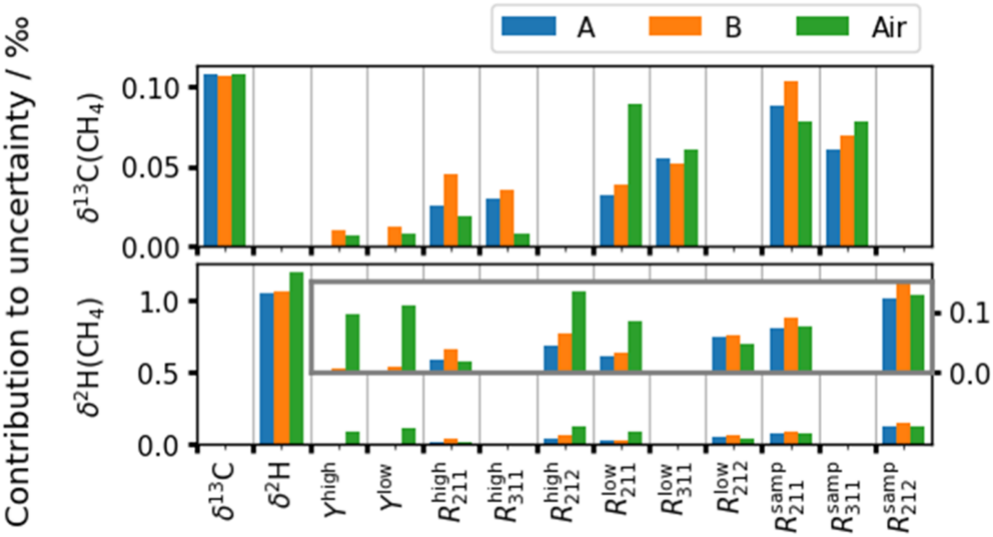
Contributions to uncertainty for δ^13^C­(CH_4_) (upper plot) and δ^2^H­(CH_4_) (lower plot) for the two CH_4_ in N_2_ samples given in Table 3 and one sample with the isotopic composition of atmospheric air. Each bar indicates the contribution to the total uncertainty. The lower plot includes an inset to show the details of the small contributions for the 11 parameters. The input parameters listed on the horizontal axis are described in the text.

The uncertainty in the calibrated isotope ratios for all samples is dominated by the contribution from the isotope ratio assigned to the CH_4_ parent used to prepare the calibration RMs. There is also negligible crossover between the isotopologue measurements: for example, the δ^13^C­(CH_4_) has a negligible sensitivity to the δ^2^H of the pure CH_4_, and the instrument responses to ^12^CH_3_^2^H. The contribution from the amount fraction of the two calibration RMs is the smallest and becomes negligible when the isotopic composition of the sample is close to that of the calibration RMs (sample A). The contribution from the spectrometer is significant and shows run-to-run variability. The contributions to the calibration uncertainty follow the magnitude of the relative uncertainty of the input quantities that are given in Table 2. The isotopic assignment of the pure CH_4_ used for the RMs has the largest uncertainty relative to the absolute value (0.28% for δ^13^C and 0.54% for δ^2^H). The relative uncertainties in the amount fraction, derived from gravimetry, and the spectrometer responses, are all around 0.02%.

The uncertainty in the reference values *r*_13_^VPDB^ and *r*_2_^VSMOW^ has not been propagated through the calibration, as these are considered as constants that define the scale and not an input parameter where the uncertainty characterizes the dispersion of values. In this calibration method, the ratios are used first in eqs 2 and 3 to calculate isotopologue amount fraction and then used in eqs 23 and 24 to calculate the sample isotope ratios. This use effectively “cancels-out” the sensitivity of the calibrated δ^13^C­(CH_4_) and δ^2^H­(CH_4_) to the reference value so that selecting a reference ratio over the uncertainty range has a negligible effect on the calibrated isotope ratio and its uncertainty.

The bars labeled “Air” in Figure 3 are calculated using the measurements of a preconcentrated sample of background air, which gives a calibrated isotopic signature of −47.32 ± 0.20‰ for δ^13^C­(CH_4_) and −90.5 ± 1.2‰ for δ^2^H­(CH_4_). Note that these measurements have not been corrected for any fractionation in the preconcentrator, so the uncertainties only include contributions from the calibration procedure. Here, the relative contributions to the δ^13^C­(CH_4_) uncertainty are similar for the fossil-sourced CH_4_ mixtures (A and B), but the δ^2^H­(CH_4_) uncertainty shows a larger contribution from the amount fraction of the calibration RMs. This indicates that the sensitivity of the calibrated isotope ratio to amount fraction uncertainty increases as the sample isotopic signature gets further from the calibration RMs. This sensitivity is investigated in the next section.

### 5.3 Contribution to Isotope Ratio Uncertainty from the RM Amount Fraction Uncertainty

The calibration RMs are prepared by gravimetry from a mixture of high-purity CH_4_ in high-purity N_2_ (BIP+, Air Products), and the sources of uncertainty in the total CH_4_ amount fraction are from the uncertainty in the balances used for weighing the gases and cylinders, the purity of the N_2_ matrix gas, and the molar mass of the components. This uncertainty is used in eq 13 and propagated to the uncertainty in isotopologue amount fraction. The relative uncertainty in the amount fraction of each RM is about 0.02%. A study of the sensitivity of the combined uncertainty to this specific source was tested over a relative uncertainty range from zero to 0.2% for sample B, and air. The results of the calculation using each uncertainty are plotted in Figure 4. These results are plotted as a dashed black line in Figure 4. In both cases, all other input quantities and their uncertainties remain fixed.

**Figure 4:**
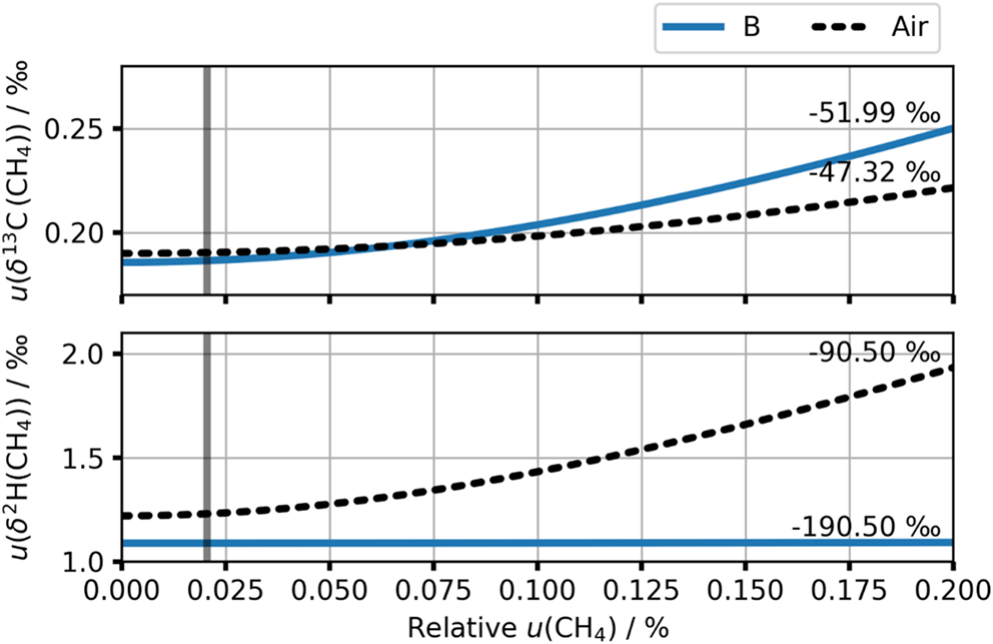
Estimated uncertainty in calibrated δ^13^C­(CH_4_) (top) and δ^2^H­(CH_4_) (bottom) under conditions of different relative uncertainty in RM amount fraction. The blue line shows the trend for sample B, and the dotted black line for a representative isotopic composition for atmospheric air. Sample A is not shown as the variation is small because the isotopic composition is identical to the calibration RMs. The lines are labeled with the isotope ratio. The vertical gray line indicates the stated relative uncertainty on the calibration RM amount fraction.

At zero relative uncertainty in the calibration RM amount fraction, there are still contributions to the combined uncertainty from the isotope ratio of the pure CH_4_ and the spectrometer; the combined uncertainty increases smoothly with the amount fraction uncertainty. The uncertainty is also lower overall when the isotopic composition of the sample is closer to that of the RMs; sample A is not shown in Figure 4 because it is produced from the same CH_4_ as the RMs, and the variation with amount fraction uncertainty is negligible. This is most clear for δ^2^H­(CH_4_), where the air is significantly enriched compared to the RMs derived from a fossil fuel source. This effect can also be seen in the bars labeled “Air” in Figure 3; the contribution from the RM amount fraction uncertainty is much larger for this sample than for those prepared from fossil-source CH_4_. The increasing uncertainty in calibrated δ^2^H­(CH_4_) is driven by the increasing RM amount fraction uncertainty and compounded by a larger sensitivity coefficient due to the difference between the isotopic signature of the sample and standard.

### 5.4 Contribution from the Covariance between Isotopologue Abundance

The uncertainty in the input quantities listed in Table 2 is uncorrelated; this is shown for the measurements in Figure 2b, the isotope ratio assignments of the CH_4_ are measured independently, and the amount fraction assignments by gravimetry are also independent. The input quantities used in the intermediate steps, however, can show significant correlation, and this is accounted for in the uncertainty propagation derived in Section 3. For example, the amount fractions of the ^12^CH_4_ and ^13^CH_4_ isotopologues in the RMs are expected to covary as they are both determined by the same pure CH_4_. For example, in the high RM (cylinder ID D914016, total CH_4_ (620.134 ± 0.106) μmol mol^–1^), the isotopologue amount fractions are (613.2 ± 0.1) μmol mol^–1^ and (6.59 ± 0.001) μmol mol^–1^ for ^12^CH_4_ and ^13^CH_4_, respectively, and the correlation coefficient calculated using the covariance from eq 14 is 0.83. The correlation coefficients between each pair of isotopologue amount fractions in the low and high RM are shown graphically in Figure 5.

**Figure 5:**
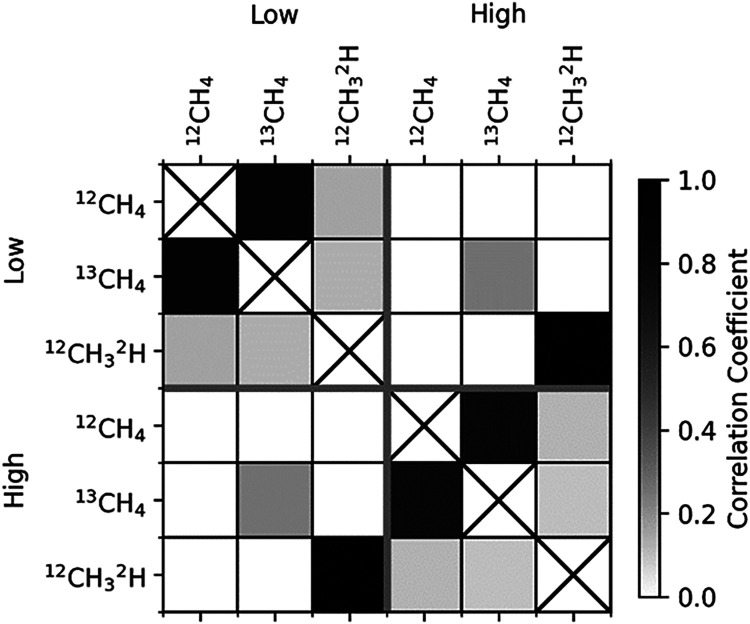
Correlation coefficient between pairs of isotopologue amount fractions in the high and low calibration RM mixtures. The plot is split into four quadrants corresponding to the two RMs. The upper left and lower right quadrants show the correlation between isotopologue amount fraction within a single RM. The lower left and upper right quadrants show the correlation between isotopologue amount fractions between the two calibration RMs.

As with the uncertainty contribution from the input quantities, it is useful to examine the relative influence of covariance by setting this term to zero for some of the uncertainty propagation. Considering the covariance between the calibrated isotopologue amount fraction *u*(*Y*_211_^samp^,*Y*_311_^samp^) and *u*(*Y*_211_^samp^,*Y*_212_^samp^) in eqs 25 and 26, there are two useful limiting scenarios to investigate: low covariance and high covariance. The calibrated isotope ratios remain the same in each example case, and the changes to the covariances only affect the uncertainties.

Uncorrelated isotopologue amount fraction corresponds to complete independence of these quantities. A low correlation between ^12^CH_4_ and ^13^CH_4_ amount fraction would arise if these were not connected through the δ^13^C isotope ratio of a common source of CH_4_ but are added separately to the RM, or the amount fraction of each isotopologue in the calibration mixtures is measured independently by an absolute method. This preparation is yet to be demonstrated for CH_4_ but has been shown to be feasible for CO_2_,^45,46^ and absolute measurements of CO_2_ isotopologue amount fraction have been demonstrated with traceability to spectroscopic constants instead of an isotope ratio scale.^47^ With the isotopologue covariance set to zero, the uncertainty of the calculated δ^13^C­(CH_4_) isotope ratio for sample B is 0.26‰ instead of 0.19‰, while the uncertainty for δ^2^H­(CH_4_) is unchanged. This shows that neglecting isotopologue covariance causes an overestimation of the uncertainty for δ^13^C­(CH_4_).

For fully correlated quantities, the product of the individual uncertainties is used as the covariance, e.g., *u*(*Y*_211_^samp^,*Y*_311_^samp^) = *u*(*Y*_211_^samp^)*u*(*Y*_311_^samp^) and *u*(*Y*_211_^samp^,*Y*_212_^samp^) = *u*(*Y*_211_^samp^)*u*(*Y*_212_^samp^). This leads to an underestimate in both isotope ratio uncertainties: 0.02 ‰ instead of 0.19 ‰ for δ^13^C­(CH_4_) and 0.9 ‰ instead of 1.1 ‰ for δ^2^H­(CH_4_).

There is also a covariance between the amount fraction of the same isotopologue between the two calibration RMs, which can be seen in the lower left and upper right quadrants of Figure 5. Neglecting this covariance leads to a small difference in both isotope ratio uncertainties: 0.17 ‰ instead of 0.19‰ for δ^13^C­(CH_4_) and 0.8 ‰ instead of 1.1‰ for δ^2^H­(CH_4_). This covariance would be zero if the low and high RMs were prepared from different CH_4_ parents.

## 6 Application to Atmospheric Measurements

The laser spectrometer is a component in the Boreas preconcentrator system, which is designed to measure δ^13^C­(CH_4_) and δ^2^H­(CH_4_) in atmospheric CH_4_. The preconcentrator system has been described in detail previously in^20,42^ . The system, in summary, collects an atmospheric air sample either in situ using a diaphragm pump or from a compressed gas cylinder. This sample passes through a trap packed with HayeSep-D adsorbent that is maintained at around −165 °C by a cryocooler. After a predetermined trapping duration, the gas flow is switched by multiposition valves to flush the trap with a slower flow of high-purity nitrogen while the trap temperature is increased. A timed sequence of valve switching first directs the more volatile species to vent, and then the spectrometer is loaded with the trap eluant composed of nominally pure CH_4_ in N_2_ at around 550 mmol mol^–1^. Finally, the less volatile species are flushed to vent, and the trap is heated to recondition it for the next run.

The calibration approach demonstrated in the preceding sections uses a pair of RMs that bracket the sample in amount fraction and then quantifies the amount fraction of the CH_4_ isotopologues. The amount fraction of CH4 in the RMs and samples is in the range of approximately 500 to 625 μmol mol^–1^, which is much higher than an ambient air sample (approximately 1.9 μmol mol^–1^). The spectrometer is coupled to a preconcentrator system that cryogenically separates CH_4_ from an air sample performing two roles: increasing the amount fraction of CH_4_ to improve the sensitivity of the spectrometer measurement and normalizing to a nitrogen matrix to remove trace gases that can interfere with the spectroscopy.^14^ Ideally, calibration of an atmospheric measurement follows the principle of identical treatment (PIT), where the air and reference gases follow identical analytical processes and have near-identical composition.^8^ Fully applying PIT here would require preconcentration of RMs, then air in subsequent measurement cycles, each of which takes more than an hour, and the uncertainty contribution from the spectrometer would be much larger due to drift, shown by the increasing Allan deviation in Figure 2a. In our atmospheric monitoring application, we use the preconcentrator system as a comparator to measure ambient air with reference to a compressed ambient air working standard that has been isotopically assigned by IRMS. This allows for correction of any fractionation effects due to the preconcentrator, as described in Rennick et al. 2021.^20,42^ The spectrometer calibration is stable with respect to drift over a sufficiently long duration for air and working standard measurement. This is shown by the standard deviation of 22 repeated measurements (about 1 day in duration) of sample B, which is 0.08‰ for δ^13^C­(CH_4_) and 0.5‰ for δ^2^H­(CH_4_).

## 7 Conclusions

An infrared diode laser spectrometer is used to measure δ^13^C­(CH_4_) and δ^2^H­(CH_4_) isotope ratios using the isotopologue calibration method, where the amount fraction of the ^12^CH_4_, ^13^CH_4_, and ^12^CH_3_^2^H isotopologues is calibrated. The benefit to this approach is that it removes the requirement for several different CH_4_ RMs with controlled isotopic signatures, e.g., selected to bracket the sample in both amount fraction and isotope ratio. Here, we use a single high-purity fossil-source CH_4_ that has been assigned δ^13^C and δ^2^H isotope ratios by IRMS and diluted in N_2_ to create two mixtures with the same isotopic signature that bracket the sample in amount fraction. The calibration approach is a multistep measurement equation, where the output quantities from one step are used as the input quantities in the next. We demonstrated calibration for measurements of a second synthetic CH_4_ sample, resulting in measurements that agree with IRMS. The spectrometer calibration is stable over a day, illustrated by the standard deviation of repeated measurements, so it is suitable for atmospheric measurements using a preconcentrator where an ambient air sample is alternated with a working standard. The multistep measurement equation has been used to derive algebraic expressions to propagate the uncertainty in input quantities—the isotope ratio of the RMs, their amount fraction, and the spectrometer uncertainty—to the calibrated isotope ratio of the sample. An uncertainty budget was constructed, which showed that the dominant contribution to the calibration uncertainty is the δ^13^C­(CH_4_) and δ^2^H­(CH_4_) assigned to the RMs. This is in line with the relative uncertainty of the input parameters: the relative uncertainties are 0.28 and 0.54% for δ^13^C­(CH_4_) and δ^2^H­(CH_4_), respectively, and between 0.01 and 0.02% for the RM amount fraction and spectrometer responses. This means that under specific conditions, some uncertainty contributions could be neglected to simplify the calculation of the combined uncertainty. This is not the general case, however, because the contribution from RM amount fraction uncertainty is larger for samples more isotopically different from the standard. This is relevant when fossil-sourced RMs are used to calibrate an ambient air sample where δ^2^H­(CH_4_) can differ by 100‰ between fossil and ambient air CH_4_.

The uncertainty propagation demonstrated the influence of correlation between isotopologue amount fractions in the combined uncertainty of the sample isotope ratio. As a single source of CH_4_ is used for two calibration RMs, the amount fraction of the ^12^CH_4_ and ^13^CH_4_ and ^12^CH_3_^2^H isotopologues is intrinsically linked. Neglecting this correlation causes an incorrect estimation of the combined uncertainty because the covariance is actually a negative term in the combined standard uncertainty for δ^13^C­(CH_4_) and δ^2^H­(CH_4_) given by eqs 25 and 26. Synthetic RMs prepared using isotopically characterized CH_4_ (with an isotopic signature that is significantly different from atmospheric CH_4_) can be used for calibration of OIRS measurements with low uncertainty if the amount fraction uncertainty is sufficiently small. The calibration needs can be largely met through maintenance of a single stock of pure CH_4_, and longer-term needs can be maintained via curation of a pure CH_4_ RM hierarchy.
